# Internet Use and Problematic Use in Seniors: A Comparative Study in Switzerland and Poland

**DOI:** 10.3389/fpsyt.2021.609190

**Published:** 2021-06-09

**Authors:** Lucien Rochat, Monika Wilkosc-Debczynska, Ludmila Zajac-Lamparska, Stéphane Rothen, Paulina Andryszak, Julie Gaspoz, Laura Colombo, Yasser Khazaal, Sophia Achab

**Affiliations:** ^1^Specialized Facility in Behavioral Addiction ReConnecte, Department of Mental Health and Psychiatry, University Hospitals of Geneva, Geneva, Switzerland; ^2^Faculty of Psychology, Kazimierz Wielki University, Bydgoszcz, Poland; ^3^Department of Mental Health and Psychiatry, Addiction Division, University Hospitals of Geneva, Geneva, Switzerland; ^4^Research Center for Statistics, Geneva School of Management and Economics, University Hospitals of Geneva, Geneva, Switzerland; ^5^Faculty of Biology and Medicine, University of Lausanne, Lausanne, Switzerland; ^6^Faculty of Medicine, WHO Collaborating Centre for Training and Research in Mental Health, University of Geneva, Geneva, Switzerland

**Keywords:** problematic internet use, seniors, well-being, impulsivity, cultural differences

## Abstract

**Background:** Seniors have been only little considered in studies examining problematic internet use and associated health issues, although they may present risk factors that make them particularly vulnerable for the development of problematic internet use.

**Objectives:** (1) To compare Internet use and problematic use among seniors in Switzerland and Poland; (2) To examine the relationships between problematic internet use, impulsivity traits and well-being as previous studies showed that internet can be used to cope with negative emotions or life dissatisfaction.

**Methods:** A cross-sectional survey conducted between June 2016 and April 2017 with 264 older internet users aged above 60 years old recruited in Switzerland (88) and Poland (176) assessing sociodemographic variables, online activities, problematic internet use, impulsivity traits and well-being.

**Results:** The two groups differed in their reported online activities in that Polish participants reported more searching for information and buying, whereas Swiss participants reported significantly greater problematic internet use than Polish participants. Finally, a multiple linear regression analysis performed on the whole sample indicated that lower well-being and being a Swiss participant were both significantly associated with greater problematic internet use, after age, gender, level of education, impulsivity traits have been controlled for.

**Discussion:** Swiss seniors showed a more problematic internet use than Polish participants who focused more in their online activities on utility use of internet. The relationships between problematic internet use and well-being suggest that older adults use internet mainly to cope with negative emotion or life dissatisfaction. Socio-cultural differences that could account for these group differences as well as difference with young adults are discussed.

## Introduction

The development and spread of the internet worldwide have been contributing to growing concerns among researchers and health professionals about problematic Internet use (PIU), prompting the World Health Organization (WHO) to examine since 2014, its public health implications (1). PIU is an umbrella construct, encompassing distress and functional impairment related to the uncontrolled use of one or more online activities in order to fulfill some specific need such as emotional coping, increased sense of belonging, improved self-esteem (2, 3). This repetitive pattern of use continues over time to an extent in which the subject experiences significant psychosocial (e.g., depression, anxiety, social isolation, academic or professional failures) and physical (e.g., sleep disorders, sedentary life-style, musculoskeletal issues) harms (1). The magnitude of the phenomenon is complex to draw due to the lack of gold standard to measure PIU and to the numerous sampling biases (e.g., youth being the main studied population and schools and universities being the most inclusion sources) (4). These issues have led to uninformative prevalence rates, to heterogeneous screeners used, to the lack of comparativeness between studies and countries and to the lack of data on additional vulnerable subgroups such as seniors (4).

PIU has been considered to result from the interplay of various psychological factors including poor self-regulation capacities, mood regulation and preference for online social interaction, with addictive properties of some online activities (e.g., gambling, gaming) and with a socio-cultural environment (2). Better understanding of PIU requires unveiling underlying psychosocial mechanisms and associated vulnerability factors, as well as examining the singularity of each of the potentially problematic online activities (i.e., gambling, gaming, social networking, porn) and specific environmental contributors (1). This is in line with the recent inclusion of Gambling and Gaming disorders among addictive behaviors category of ICD-11 (5) and with WHO ongoing efforts to develop a gold standard for each of these disorders, considering the vector Internet a facilitating factor only.

Although older adults constitute a part of the population with the lowest level of Internet use (“the digital divide”), Internet access and use among older adults is progressively increasing in Europe and worldwide. For instance, in Switzerland, 10% of older adults aged 60–69 and 1% above 70 were using the internet in 2000 growing to respectively 73 and 45% in 2017 (6). In the US, internet use among adults over 65 years old went from 12% in 2000 to 67% in 2016 (7). In Poland, the percentage of older adults using the internet increased to 77.5% (1.6% more than the previous year) in 2018 (8). A continuous increase of the internet use worldwide can be expected not only because of marketing coverage but also because of the current young adults using the internet will constitute the older users in the future (9).

While the proportion of older adults over 60 continuously increases worldwide, the question arises about the use and potential misuse of the internet in the seniors. The consequences of the internet use in seniors remain a controversial issue though (10). Indeed, on the one hand, some studies underlined the benefits of the internet use in the seniors inasmuch as internet can increase the number of contacts with family and friends, promote the development of new relationships, reduce social isolation and loneliness (11), provide entertainment, maintain social involvement, empowerment and experience of control which in turn increases mental health, life satisfaction and the quality of life (12). The use of internet for telemedicine might also be beneficial for the seniors (13). Finally, internet use in midlife has been significantly associated with a lower dementia incidence in a longitudinal study (14). On the other hand, some researchers stressed that Internet use was significantly associated with decreased time spent with friends and local social networking, which in turn increased loneliness, promotes isolation and marginalization as well as decreased quality of life (15–17). It seems thus likely that a subgroup of seniors presents PIU and associated negative consequences. In comparison to youths and young adults however, seniors have been only little considered in studies examining PIU and associated health issues (1), although they may present risk factors such as a sedentary life style, social isolation/loneliness, psychiatric and neurological disorders (e.g., depression, cognitive impairments) (9). Moreover, economic problems due to the reduction of the income can make them particularly vulnerable for the development of PIU, particularly online gambling disorder (9). Another unresolved question regards the cultural differences for Internet use and misuse and the need for more comparative research on PIU in older adults from Europe and other parts of the world (18, 19).

The first aim of the current study was thus to compare online behaviors among seniors in Switzerland and Poland. The two European countries have a comparable rate of Internet use among seniors (6, 8), and they represent different parts of the continent, which have been shaped by different socio-cultural environment (e.g., religious, economic, political and social systems). A second objective was to examine the association between PIU, impulsivity traits and emotional well-being in seniors from both countries. As frequently stressed in youth and young adults, lack of self-regulation and maladaptive emotional coping seem to play a role in the development of PIU, as internet can be used to cope with difficulties and negatives emotions or life dissatisfaction (20).

## Materials and Methods

### Sample

Subjects consisted of French or Polish-speaking adults internet users aged 60 years or older recruited from June 2016 to April 2017. A total of 374 subjects began the survey (237 Polish and 137 Swiss) of whom 264 (176 Polish and 88 Swiss) were retained for the analyses after removing participants with too many missing data and/or participants reporting no internet use. Subjects' age ranged from 60 to 91 years (*M* = 67.0, *SD* = 5.9) in the Swiss sample (33% women), and from 60 to 84 (*M* = 65.9, *SD* = 5.9) in the Polish sample (57% of women). Regarding, the level of education, 59% of Swiss subjects report having completed higher studies (university level) compared to 22% in the Polish sample.

### Questionnaires and Procedure

The Swiss sample was recruited through advertisement in seniors' university or clubs and researchers' private networking (e.g., acquaintances, neighborhood) and were invited to take part to an online survey. The Polish sample was recruited through researchers' private networking (e.g., acquaintances, neighborhood), by applying a snowball sampling procedure (e.g., study participants recruited further participants from among their acquaintances) and participants were administered the survey face-to-face in a paper-and pencil format.

Participants answered items assessing sociodemographic variables (age, gender, level of education), questions on type of online activities (yes/no format) following by three questionnaires assessing compulsive internet use, impulsivity traits and psychological well-being, respectively.

### Compulsive Internet Use Scale

The CIUS (21) consisted of the 14 items scored on a 5-point Likert scale from 0 (never) to 4 (very often). The items of the CIUS assess several features of addiction such as loss of control (e.g., “Do you find it difficult to stop using the Internet when you are online?”), preoccupation regarding Internet use (e.g., “Do you think about the Internet, even when not online?”), withdrawal symptoms (e.g., “Do you feel restless, frustrated, or irritated when you cannot use the Internet?”), coping or mood modification (e.g., “Do you go on the Internet when you are feeling down?”) and conflict, including inter- and intrapersonal conflict (e.g., “Do others say you should use the Internet less?”). This questionnaire showed fair psychometric properties in various languages, including French and Polish (22). The higher the score, the greater the compulsive use of the internet. In the current study, the internal consistency (Cronbach's alpha) of the scale is 0.92.

### Short Urgency-Premeditation-Perseverance-Sensation Seeking-Positive Urgency Impulsivity Behavior Scale

The S-UPPS-P scale (23) is a 20-item self-report measure that assesses five dimensions of impulsivity: positive urgency (e.g., “When I'm happy, I often can't stop myself from going overboard”), negative urgency (e.g., “When I feel rejected, I often say things that I later regret”), (lack of) perseverance (e.g., “I am a person who always gets the job done”), (lack of) premeditation (e.g., “I usually make up my mind through careful reasoning”), and sensation-seeking (e.g., “I welcome new and exciting experiences, even if they are a little frightening or unconventional”). Items are rated on a 4-point scale ranging from 1 (I agree strongly) to 4 (I disagree strongly), with higher scores indicating greater impulsivity. This scale has good internal consistency, test–retest stability, predictive validity and has been validated in various languages (23). Higher score reflects greater impulsivity. In the current study, the internal consistency (Cronbach's alpha) of the subscales range from 0.63 to 0.86.

### Short Happiness and Depression Scale

The SDHS (24) consists of six items assessing happiness (e.g., “I feel happy”) or depression (e.g., “I feel dissatisfied with my life”). Scale items are scored from 1 (never) to 4 (often). We reversed depression items so that higher scores reflected positive mood. This reverse scoring has been used in the original version of the scale as well as in its shorter form and was shown to be valid (24). The scale thus provides a single continuous measure of the depression–happiness continuum. The SDHS has shown good internal consistency, test–retest reliability, and convergent and discriminant validity in various languages. In the current study, we used a French and, respectively, Polish translation of the SDHS. The scale was translated from English into French and then back-translated from French into English (25). The same procedure was used in Polish. Although there is no validation study of this scale in French or Polish, this scale has been sucessfully used in other studies and showed a good internal consistency (25). In the current study, the internal consistency of the scale (Crobach's alpha) is 0.78.

### Statistical Analyses

First, group comparisons on socio-demographic variables, type of online activities and scores on the CIUS, SDHS and S-UPPS-P were performed using Welch two sample *t*-test for continuous variables and Pearson's Chi-squared test with Yates' continuity correction for dichotomous variables. Second, correlation analyses were performed on the whole sample to examine the association between all the variables of interest using Spearman's Rho non-parametric tests. Third, multiple linear regression analyses were used to examine the relationships between PIU and socio-demographical variables (age, gender, level of education), group (Swiss vs. Polish participants), impulsivity traits as well as psychological well-being on the whole sample. All analyses were two-tailed, with a significance threshold set at 0.05. Regarding missing data, when one item was missing, a person-mean imputation was performed. When more than one item was missing, the subject has been removed from the analysis. Thus, the sample size decreases from 88 to 66 and from 176 to 171 participants in the correlation and regression analyses in the Swiss and Polish sample, respectively, because participants with too many missing data (>1) were excluded from the analyses.

### Ethics

The study was carried out in accordance with the Declaration of Helsinki and was approved by the local Ethical Committees in both countries.

## Results

### Group Comparisons

#### Demographic Data

Group comparisons indicate that the proportion of male and participants with an education at the university level was significantly greater in the Swiss sample than in the Polish sample whereas age was not significantly different between the groups (Table 1).

**Table 1:** Demographical data.

	**Swiss sample (*N* = 88)**	**Polish sample (*N* = 176)**	**Statistics**	
**Variables**	**M (SD)**	**M (SD)**	***t* (174.9)**	***p*-value**
Age (years)	67.0 (5.90)	65.9 (5.90)	1.40	0.17
	**%**	**%**	***χ^2^(1)***	***p*****-value**
Sex (male/female)	67/33	43/57	12.53	<0.001
Educational level (university/no university)	59/41	22/78	32.235	<0.001

#### Online Activities, CIUS, S-UPPS-P, SDHS

First, social media use and searching for information on the Internet were the two more frequently reported online activities by both groups. Group comparisons indicate that the proportion of participants who use internet for searching for information and buying was significantly greater in the Polish sample than in the Swiss sample. No other online activities reached statistical significance. Second, Swiss seniors reported a significantly greater score on the CIUS as well as on lack of premeditation and sensation seeking than the Polish participants, whereas the Polish seniors showed higher scores on negative urgency. No other comparisons reached statistical significance (Table 2).

**Table 2:** Group comparisons on type of online activities, CIUS, S-UPPS-P, SDHS scores.

	**Swiss sample**	**Polish sample**		**Statistics**
**Variables (dichotomic)**	**%**	**%**	***χ^2^***	***p*-value**
Social media (yes/no)	59/41	48/52	2.60	0.11
Information (yes/no)	36/64	68/32	22.14	<0.001
Videos (yes/no)	17/83	9/91	2.86	0.09
Gaming (yes/no)	11/89	7/93	1.05	0.31
Email (yes/no)	11/89	14/86	0.20	0.65
Professional (yes/no)	3/97	6/94	0.46	0.50
Buying (yes/no)	2/98	13/87	6.77	<0.01
**Variables (continuous)**	**M (SD)**	**M (SD)**	***t***	***p*****-value**
CIUS total score	10.5 (8.8)	8.0 (8.6)	2.01	<0.05
S-UPPS-P Negative urgency	8.7 (2.6)	9.6 (2.9)	−2.44	<0.05
S-UPPS-P Positive urgency	10.0 (2.2)	9.9 (2.6)	0.43	0.67
S-UPPS-P Lack of premeditation	7.7 (2.5)	7.0 (2.0)	2.00	<0.05
S-UPPS-P Lack of perseverance	7.1 (2.8)	6.6 (2.4)	1.31	0.19
S-UPPS-P Sensation seeking	9.3 (2.3)	8.0 (2.7)	3.74	<0.001
SDHS	13.3 (3.2)	12.6 (3.2)	1.44	0.15

*CIUS, Compulsive Internet Use Scale; S-UPPS, Short UPPS Impulsivity Behavior scale; SDHS, Short Happiness and Depression Scale*.

### Correlation Analysis

Correlation analysis between CIUS, socio-demographical variables, impulsivity traits and emotional well-being using Spearman's Rho on the whole sample indicates that PIU as assessed by the CIUS was significantly associated with a lower well-being on the SDHS (ρ = −0.18, *p* <0.01), a greater level of negative urgency (ρ = 0.19, *p* < 0.01), lack of premeditation (ρ = 0.17, *p* < 0.01), as well as lack of perseverance (ρ = 0.15, *p* < 0.05). No other correlation reached statistical significance, including the correlation between level of education and CIUS (ρ = 0.04, *p* = 0.59).

### Multiple Linear Regression

The multiple linear regression analysis performed on the whole sample indicates that a lower psychological well-being as well as being a Swiss participants were both significantly associated with a higher score on the CIUS, while age, level of education, gender and dimensions of impulsivity were controlled for, *F*_(10, 221)_ = 3.01, *p* < 0.01, *adjR*^2^ = 0.08 (Table 3).

**Table 3:** Multiple linear regressions by groups on the CIUS total score (*N* = 237).

**Variables**	**Estimate**	**Std.Err**	***t*-value**	***p-*value**
Age	−0.07	0.10	−0.70	0.49
Sex (male)	0.87	1.13	0.77	0.44
Level of education (university)	−0.63	1.26	−0.50	0.62
Group (Swiss)	3.14	1.40	2.24	<0.05
S-UPPS-P-Negative urgency	0.34	0.26	1.31	0.19
S-UPPS-P-Positive urgency	0.00	0.31	−0.01	0.99
S-UPPS-P-Lack of premeditation	0.02	0.36	0.05	0.96
S-UPPS-P-Lack of perseverance	0.08	0.31	0.25	0.80
S-UPPS-P-Sensation seeking	0.05	0.24	0.20	0.84
SDHS	−0.71	0.18	−3.90	<0.001

*S-UPPS, Short UPPS Impulsivity Behavior scale; SDHS, Short Happiness and Depression Scale*.

## Discussion

This study aimed to compare Internet use and problematic use among seniors in Switzerland and Poland a well as to examine the relationships between PIU, impulsivity traits and psychological well-being. The main results showed that the two groups partly differed in their reported online activities in that Polish participants reported more searching for information and purchase compared to the Swiss sample. However, Swiss participants reported significantly greater PIU than the Polish participants. Finally, lower well-being and being Swiss participants were both significantly associated with PIU while age, gender, level of education, impulsivity traits were controlled for.

First, regarding Internet use, our results corroborate previous studies stressing that online activities have evolved over time and now focus more on social media and search for information. Indeed, internet constitutes a way to maintain or increase contacts with friends or family members, and this may be especially relevant for people with physical disabilities or when family members or friends live abroad. In this context, virtual communication could potentially prevent or reduce seniors' social isolation (26). The results also indicate that the Internet can enable a better access to information such as health-related information, news or access to medicine (13). Group differences stressed between Swiss and Polish seniors could be partly explained by sociodemographic differences (gender and education oportunities) inasmuach as the Polish sample is made of 2/3 by women aged over 60, and <1/4 of this sample reached university degrees. In this context, searching information online may be considered an attempt to close anonymously, affordably and fastly some knowledge gap, especially in the Polish sample. In addition, more frequent purchase on the Internet in the Polish sample may be associated with the economic status inasmuch as the participants could search for instance for sales or cheaper products and/or for products that can be found abroad only. On the whole, the group comparisons indicate that the main group difference resides in a more utilitarian use of Internet (information search and purchasing) by Polish seniors that cannot be accounted for by age, probably to overcome daily obstacles. Tentatively, these differences might also be due to an early stage of digital penetration in that when the Internet becomes more available for seniors, it mainly serves practical daily needs and purposes, whereas with continued availablity it evolves into a more leisure-oriented use. By contrast, young adults might be initially more driven by looking for fun activities online (e.g., gaming) than for practical activities (27). Further studies should more specifically examine the influence of some sociodemographical, geographical and cultural variables (e.g., economic status, religion, urban vs. rural area), as well as expertise with the Internet on types of online activities among seniors.

Second, Swiss seniors reported more PIU than Polish participants. Although in recent years access to the Internet has become increasingly widespread, it has started later for seniors in Poland (28). Indeed, PIU probably requires greater familiarity with the Internet, and ease and flexibility of movement on the web and may then become problematic if vulnerability factors are met. In this perspective, the significant relationships between PIU and psychological well-being suggest that participants use Internet mainly to cope with negative emotion or life dissatisfaction. Indeed, participants with dysphoric mood may search for virtual environments where they can momentarily decrease their negative feelings inasmuch as they can find an infinite amount of distracting online activities including social media or videos (29, 30). However, in contrast to findings obtained in young adults (31), impulsivity traits were not significant predictors of PIU in seniors in our study. This result suggests that Internet may be use to cope with negative emotion, but that there is no addictive use pattern for most seniors. More generally, the mean level of PIU as assessed by the CIUS is much lower in our sample (10.5 and 8.0 for the Swiss and Polish seniors, respectively) than in a large sample of young adults (mean 24.63 years old) where PIU mean score was 16.03 (22). Taking into account the type of online activities could account for this discrepancy between young adults and seniors inasmuch as some activities carried out by the formers are potentially much more addictive than those carried out by the latters. In particular, online video gaming disorder is a common problem in adolescents and young adults and has been frequently associated with elevated level of impulsivity (32). In our study, gaming was only reported by 11 and 7% of the Swiss and Polish participants, respectively.

Some limitations to the study must be acknowledged. Indeed, the study is cross-sectional, and further research is required to longitudinally confirm the association between impulsivity traits, psychological well-being and/or age in problematic internet use. Furthermore, the sample is self-selected, limiting the generalizability of results to the entire population of older adults using the internet (33) and the representativeness of each sample could be impacted by the different ways the participants were recruited. Moreover, the study relies exclusively on self-reports which have been associated with various biases (e.g., social desirability and lack of insight). We also had a small sample size especially in the Swiss sample. Conclusions should thus be drawn only tentatively.

## Conclusion

In conclusion, this is the first study to examine excessive Internet use in a sample of seniors. The results also underline the necessity to take into account cultural and socio-demographic background when examining internet use and misuse. The results also indicate that PIU may be present in a subgroup of seniors Internet users and that Internet can be used to cope with negative emotions or life dissatisfaction. Finally, as Internet users are constantly growing in number among seniors, further research are needed to better appraise the psychological, biological, social and cultural underpinnings of PIU in older adults, and to promote effective prevention strategies and tailored treatment.

## Data Availability Statement

The raw data supporting the conclusions of this article are available by the corresponding author, without undue reservation.

## Ethics Statement

The studies involving human participants were reviewed and approved by Ethical Committee of Faculty of Psychology, Geneva, Switzerland. The patients/participants provided their written informed consent to participate in this study.

## Author Contributions

SA, LR, MW-D, LZ-L, and SR designed the study, ran the research, discussed the results, and drafted the manuscript. YK participated to the design of the study. JG participated to ethical submission in Switzerland. PA participated to the study design and recruitment procedure for the polish sample. LC participated to the statistical analysis. All authors contributed to the article and approved the submitted version.

## Conflict of Interest

The authors declare that the research was conducted in the absence of any commercial or financial relationships that could be construed as a potential conflict of interest.
